# Early intervention of tau pathology prevents behavioral changes in the rTg4510 mouse model of tauopathy

**DOI:** 10.1371/journal.pone.0195486

**Published:** 2018-04-06

**Authors:** Xiaohai Wang, Karen Smith, Michelle Pearson, Anna Hughes, Mali L. Cosden, Jacob Marcus, J. Fred Hess, Mary J. Savage, Thomas Rosahl, Sean M. Smith, Joel B. Schachter, Jason M. Uslaner

**Affiliations:** Merck Research Laboratories, West Point, Pennsylvania, United States of America; Nathan S Kline Institute, UNITED STATES

## Abstract

Although tau pathology, behavioral deficits, and neuronal loss are observed in patients with tauopathies, the relationship between these endpoints has not been clearly established. Here we found that rTg4510 mice, which overexpress human mutant tau in the forebrain, develop progressive age-dependent increases in locomotor activity (LMA), which correlates with neurofibrillary tangle (NFT) pathology, hyperphosphorylated tau levels, and brain atrophy. To further clarify the relationship between these endpoints, we treated the rTg4510 mice with either doxycycline to reduce mutant tau expression or an O-GlcNAcase inhibitor Thiamet G, which has been shown to ameliorate tau pathology in animal models. We found that both doxycycline and Thiamet G treatments starting at 2 months of age prevented the progression of hyperactivity, slowed brain atrophy, and reduced brain hyperphosphorylated tau. In contrast, initiating doxycycline treatment at 4 months reduced neither brain hyperphosphorylated tau nor hyperactivity, further confirming the relationship between these measures. Collectively, our results demonstrate a unique behavioral phenotype in the rTg4510 mouse model of tauopathy that strongly correlates with disease progression, and that early interventions which reduce tau pathology ameliorate the progression of the locomotor dysfunction. These findings suggest that better understanding the relationship between locomotor deficits and tau pathology in the rTg4510 model may improve our understanding of the mechanisms underlying behavioral disturbances in patients with tauopathies.

## Introduction

Intracellular accumulation of neurofibrillary tangles (NFTs) is the hallmark of a family of neurodegenerative diseases known as tauopathies, including Alzheimer’s disease (AD), frontotemporal dementia (FTD), corticobasal degeneration (CBD), Pick’s disease, and progressive supranuclear palsy (PSP). AD is the most common tauopathy, and the amount of NFTs correlates with the severity of cognitive dysfunction [1,2]. In frontotemporal dementia with parkinsonism type-17 (FTDP-17), mutations in tau can initiate the disease, suggesting that tau is causally related to the neurodegeneration and dementia that is observed [3]. Most tau mutations affect tau microtubule binding or result in alternative splicing of tau mRNA [4]. The discovery of specific tau mutations has facilitated the generation of mouse models of tauopathy, which has allowed for pre-clinical evaluation of novel therapeutic strategies to treat these diseases [5].

Behavioral impairments have been observed in most of the tau transgenic models [5]. Among these impairments, motor deficits are especially apparent when the prion promoter (PrP) or Thy1 promotor were used to overexpress mutant tau [6–11]. However, it is unclear whether the motor deficits that have been observed are due to pathology in the forebrain or brain stem and spinal cord, given that PrP and Thy 1 are largely expressed in the brainstem and spinal cord [6–11]. The rTg4510 mouse model was developed in an effort to restrict tau pathology to the forebrain structures. In this model, mutant tau expression is under control of the Ca^2+^-Calmodulin kinase IIα (CaMKIIα) promotor, which is restricted to forebrain structures. Consistent with the forebrain expression of tau pathology in rTg4510 model, the initial model characterization did not show robust motor differences compared to wild type (WT) mice [12,13]; however, more recent reports indicate that rTg4510 mice also exhibit locomotor changes in the open field test [14–16]. Although the locomotor dysfunction is an unwelcome phenotype in transgenic animal models as it often confounds interpretation of cognitive tests or other behavioral measures, locomotor activity may be interesting in its own right given that wandering behavior, has also been observed in dementia patients, including AD [17,18]. In addition, behavioral disturbances in FTD patients are often associated with disinhibition, resulting in impulsivity and hyperactivity [19]. Thus, understanding the relationship between hyperactivity and tau pathology in the rTg4510 mouse model may provide insight regarding behavioral disturbances in AD and FTD.

Tau is subject to many post-translational modifications, including phosphorylation, acetylation, ubiquitination, nitration, and O-GlcNAcylation [20,21]. Tau O-GlcNAcylation is a reversible modification of serine or threonine residues by O-linked N-acetyglucosamine (O-GlcNAc), which is regulated by two enzymes in mammalian cells. O-GlcNAc transferase (OGT) catalyzes the addition of O-GlcNAc, and O-GlcNAcase (OGA) catalyzes the removal of O-GlcNAc [22]. Growing evidence indicates that decreases in tau O-GlcNAcylation may contribute to tau pathology in AD [23], whereas increasing tau O-GlcNAcylation with Thiamet G, a selective OGA inhibitor, reduces the formation of tau aggregates and protects against neuronal degeneration in a mouse model of tauopathy [24].

Here we systematically characterized the hyperactivity phenotype in the rTg4510 line, and demonstrated that the age-dependent increase in locomotor activity closely correlates with the progression of brain tau pathology and brain atrophy in this animal model. To determine whether pharmacologically modulating the progression of tau pathology could alter the development of hyperactivity, we treated the rTg4510 mice with either doxycycline, which suppress the mutant tau expression, or Thiamet G, which has been shown to ameliorate tau pathology in tau transgenic models [24]. We found that both doxycycline and Thiamet G treatment, when started at 2 month of age, completely prevented the progression of hyperactivity, reduced brain tau pathology, and ameliorated the associated brain atrophy.

## Materials and methods

### Mice

This study was conducted in strict accordance with the National Research Council’s Guide for the Care and Use of Laboratory Animals. The protocol was approved by Merck Institutional Animal Care and Use Committee. The rTg4510 mouse line overexpressing human 4R0N tau with P301L mutation was licensed from the Mayo Clinic and was bred and maintained at Taconic, Hudson, NY. Briefly, the mice were generated by crossing the activator line (129S6 strain) expressing tetracycline-controlled transcriptional activator (tTA) driven by the CaMKIIα promoter and the responder line (FVB/N strain) expressing human 4R0N tau with P301L mutation driven by tetracycline-operon responsive element (TRE) as described in [12]. Only the F1 generation with 50% 129S6 strain and 50% FVB/N strain was used in this study. The mice were housed under standard laboratory conditions of controlled temperature, humidity, and lighting [12-hour light: 12-hour dark; lights on at 6:00 AM] at Merck facility at least 1 week before undergoing behavior testing to allow acclimation.

### Drug administration

For chronic doxycycline treatment, rTg4510 mice and wildtype litermate controls were fed chow containing doxycycline (1g/kg, Teklad #7012) *ad libitum* from either 2 to 6 months of age or 4 to 8 months of age. Vehicle groups were fed normal mouse chow without doxycycline. For chronic treatment with Thiamet G, mice received daily oral gavage dosing of Thiamet G at 500mg/kg in water from 2 to 4 months of age. Thiamet G was synthesized at Merck according to [25] Vehicle group received daily oral gavage dosing of water (5 ml/kg body weight) throughout the study.

### Spontaneous LMA

Spontaneous LMA was measured using digital activity monitors (Kinder Scientific, Poway, CA). Each apparatus consisted of a clear plexiglass box (7 X 15 in.) placed within the activity monitor. All LMA tests were performed during light cycle between 9:00 AM and 11:00 AM. A potential influence of altered circadian rhythm on LMA was not investigated in the current study. During the test, mice were free to ambulate and to rear for 30 min. LMA was detected by infrared photo beam breaks, and the number of beam breaks was recorded and analyzed by a computer connected to the apparatus. LMA was recorded in 10 min segments during the 30 min test session. Activity chambers were cleaned with a diluted alcohol solution after each test run to eliminate residual odor cues.

### Mouse CSF collection

Mice were euthanized with CO_2_ and placed on the stereotaxic frame with head tilted down ~80 degrees. The skin and muscle were carefully removed above the cisterna magna, and dura matter was exposed under a surgical microscope. A small opening on dura matter was made with the tip of a 30 G needle, and the CSF was drawn from the cisterna magna with a 20 μL pipettor. CSF samples were quickly transferred to a 500 μl eppendorf tube and frozen on dry ice.

### Protein extraction, Western blot, and AlphaLISA

Mice were euthanized with CO_2_, and their brains were dissected quickly. After removing the olfactory bulbs and the cerebellum, the forebrains were weighed and cut in-half sagitally. The left hemiforebrain was fixed in 10% formalin for histology, and the right hemiforebrain was immediately frozen on dry ice and kept in -80°C for biochemical analysis.

Hemiforebrain tissues were homogenized using 5 mm Qiagen metal beads with TissuLyzer (Qiagen) in 900 μL Phospho Safe extraction buffer (EMD/Novagen) plus Complete EDTA-free Protease Inhibitor Tablet (Roach). The brain homogenates were spun down at 14,000 g for 15 min at 4°C and the supernatants were collected and stored in -80°C for further analysis.

For Western-blot analysis, the supernatant containing 5 μg of protein was loaded onto 16-well 8% Bis-Tris Midi gels (Invitrogen), and run at 160 V for 75 min. After transferring the protein onto nitrocellulose membrane (Invitrogen) using the iBlot system (Invitrogen), the membrane was blocked with Li-Cor blocking buffer for 1 h at room temperature. Primary antibodies including HT7 (1:1000; Pierce, MN1000), PHF6 (1:1000; Covance, SIG-39430), and β-actin (1:5000; Abcam, ab8227) were added to the blocking buffer for overnight incubation at 4°C. Blots were washed 4 times for 5 min each with Tris-buffered saline/0.1% Tween-20 (TBS-T), and the secondary antibodies, goat anti-mouse 800 CW (1:15,000; Li-Cor) and goat anti-rabbit 700CM (1:15,000; Li-Cor) were added to the blocking buffer for 45 min incubation at room temperature. Blots were washed 4 times for 5 min each with TBS-T before being scanned with the Odyssey system (Li-Cor). Protein intensity was corrected for background and normalized to the β-actin signal.

To improve throughput and better quantify hyperphosphorylated 64 KD tau levels in brain and CSF, we developed AlphaLISA-based sandwich immunoassays. Briefly, tau protein was captured by a biotin-labeled monoclonal mouse anti-human tau antibody, PHF6 (Covance, SIG-39430) or BT2 (Thermo Scientific, MN1010B), which were then bound to streptavidin-coated donor beads (Perkin Elmer, 6760002). Detection was accomplished by monoclonal mouse anti-human total tau antibody, HT7 (Pierce, MN1000) conjugated to the acceptor beads directly (Perkin Elmer). Assay reactions (100 μL) were carried out in 96-well ½ Area OptiPlates (Perkin Elmer, 6005569) that contained 10 μL of analyte at specified protein concentration for brain homogenates or 1.2 μL of analyte plus 8.8 μL of AlphaLISA ImmunoAssay Buffer (Perkin Elmer, AL000F) for mouse CSF samples, 20 μL of biotin-labeled capture antibodies (1 nM final concentration), 20 μL detection antibody-conjugated acceptor beads (10 μg/ml final concentration). After 2 hr incubation at room temperature, 50 μL of streptavidin donor beads were added under subdued light condition (40 μg/ml final concentration) and the reaction was incubated at room temperature for 1 h with gentle shaking. The fluorescent signal was detected on an Envision Plate Reader with AlphaScreen standard settings (Perkin Elmer).

WB analysis with human total tau antibody HT7 in the low-speed spin (14,000 g) supernatants of 5-month-old rTg4510 mouse brain revealed both normal human tau protein (~55KD species) and pathologically hyperphosphorylated 64KD tau protein (S1 Fig). In contrast, the PHF6 antibody only detected hyperphosphorylated 64KDa tau (S1 Fig), which had a strong correlation with the 64KD tau under HT7 detection (r = 0.9, p < 0.0001, S1 Fig). This observation is consistent with other findings that some phosphoepitope-specific antibodies, such as AT8, detect hyperphosphorylated 64KD tau species but not the normal ~55KD tau protein under the condition used here [26,27]. Therefore, we conjugated the HT7 and PHF6 antibodies to the acceptor and donor beads, respectively, in the alphaLISA assay to detect the hyperphosphorylated 64KD tau species in low-speed spin supernatants from rTg4510 brain. As expected, PHF6 alphaLISA signal strongly correlated with both PHF6 WB signal (r = 0.93, p < 0.0001, S1 Fig) and the 64KD tau signal with HT7 WB detection (r = 0.91, p < 0.0001, S1 Fig). Additionally, the analysis of the relationship between the PHF6 alphaLISA signal and the NFT counts in entorhinal cortex also revealed a strong correlation (r = 0.87, p < 0.0001, S1 Fig). Overall, these data demonstrate that PHF6 alphaLISA can reliability detect pathologically hyperphosphorylated 64KDa tau species in rTg4510 brain.

### Histology

Hemiforebrains were immersion fixed for 48 hours in 10% neutral buffered formalin in 4°C, blocked coronally into four 3mm slabs using a brain matrix, and processed and embedded into paraffin. Immunohistochemistry was performed on 5 μm coronal sections using automated immunostainers (Ventana Medical Systems, Discovery XT) with nY-29 (Covance, 1:200) mouse monoclonal antibody and counterstained with hematoxylin. After coverslipping, slides were digitized using a slide scanner (Aperio ScanScopeXT, Leica) and numbers of NFT positive neurons were counted in the entorhinal cortex region using image analysis software (Aperio ImageScope) after manual determination of the region of interest.

### Statistics

In all figures, data are presented as means ± SEM. All statistics were performed with the software Prism (GraphPad). A p value of <0.05 was considered significant. The statistical treatment of each data set is described individually in the results.

## Results

### Increased locomotor activity in rTg4510 mice

**Fig 1:**
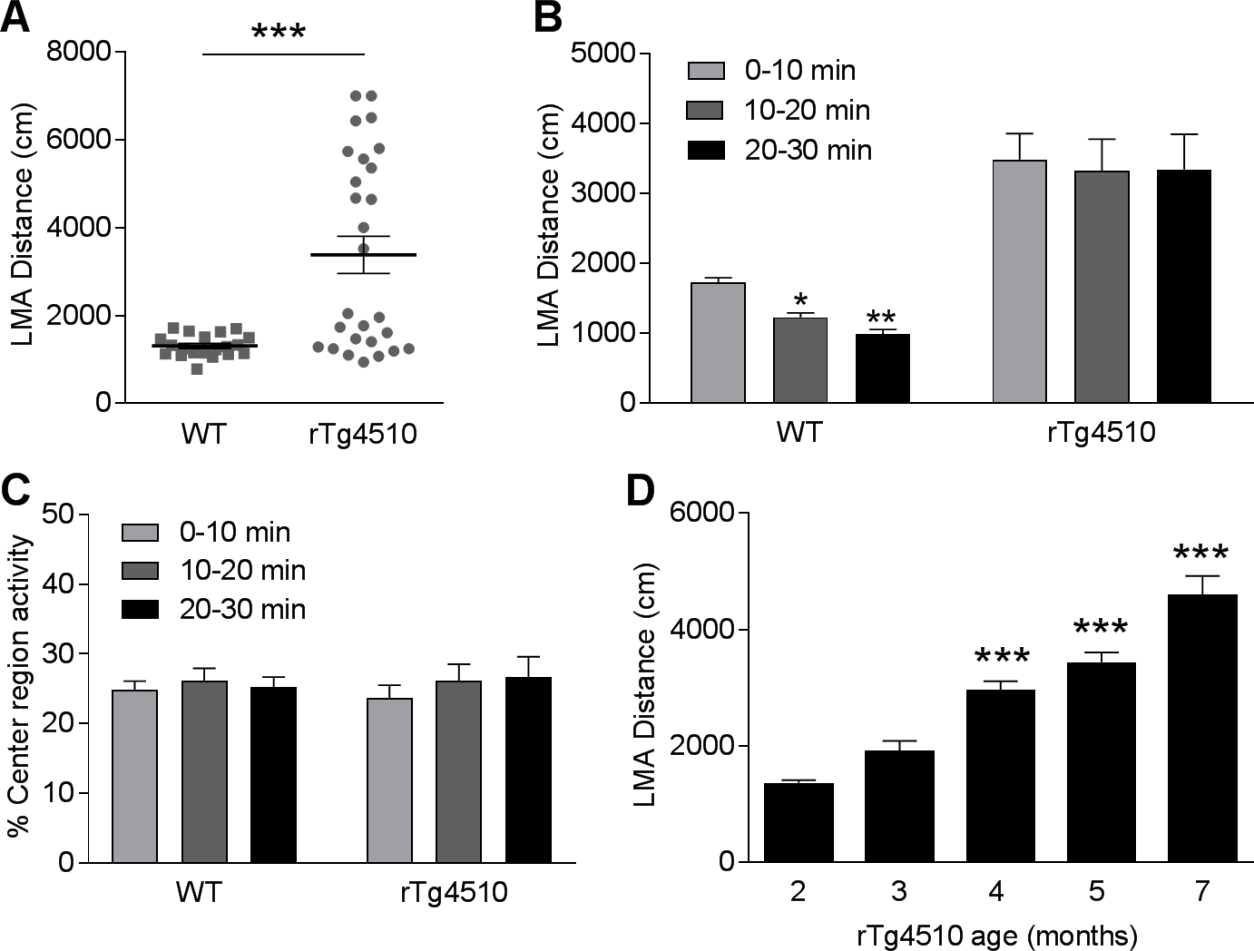
rTg4510 mice exhibit increased LMA in an age-dependent manner. (A) Averaged travel distance during a 30 min LMA test revealed increased LMA in 5-month-old rTg4510 mice compared to the WT littermates. (***p<0.001, t-test, n = 20 WT and 27 rTg4510 mice). (B) Time course distance traveled in 10-min bins. (* p<0.05, **p<0.01 compared to the first time bin, two-way ANOVA followed by Bonferroni post hoc test, n = 20 WT and 27 rTg4510 mice). (C) Five-month-old rTg4510 mice displayed similar percentage of center region activity as the WT littermates. (D) rTg4510 mice exhibited age-dependent increase in LMA. (***p<0.001 compared to 2-month-old rTg4510 mice, one-way ANOVA followed by Bonferroni post hoc test; n = 120, 124, 229, 190, and 36 per group for rTg4510 mice at 2, 3, 4, 5, 7 months of age, respectively).

Locomotor function was evaluated by measuring the spontaneous locomotor activity (LMA) of rTg4510 mice and age-matched WT littermates in an open-field box. We first examined the LMA of rTg4510 mice at 5 months of age, the age at which the rTg4510 mice develop robust tau pathology and brain atrophy in forebrain structures [12,28]. During the 30 min LMA test, the 5-month-old rTg4510 mice showed a significant increase in LMA compared with the WT littermates (Fig 1A, 158.1% increase, p < 0.001, t-test). We next analyzed the distance traveled as a function of 10-min bins during the 30 min LMA test. Two-way ANOVA indicated significant effects of genotype (Fig 1B, F(1, 45) = 17.4, P < 0.001) and time (Fig 1B, F(2, 90) = 5.4, P < 0.01) on LMA distance. Post hoc analysis showed that the distance traveled by WT mice was significantly reduced by 28.9% (p < 0.05) and 42.5% (p < 0.01) during the 2^nd^ and the 3^rd^ 10-min bins, respectively, as compared to the 1^st^ 10-min bin. However, rTg4510 animals did not exhibit a reduction in LMA over time (p > 0.05). These data suggest that WT but not rTg4510 mice habituated to the novel environment over the 30 min LMA test (Fig 1B).

To understand if increased LMA is associated with anxiety-like behavior in rTg4510 mice, we measured the percentage of activity in the center region of the open field box (center/center + peripheral) (Fig 1C). Mouse with anxiety-like behavior has been shown to avoid the center region of open field [29]. In this assay, WT mice and rTg4510 exhibited similar percentage of center region activity over the 30 min LMA test (Fig 1C) suggesting lack of anxiety-like behavior in rTg4510 mice at 5 months of age.

Given that rTg4510 mice develop age-dependent progression of tau pathology and brain atrophy [12,13,28], we next determined if the increase in LMA also progresses in an age-dependent manner. We measured the LMA of rTg4510 mice in different age groups and found that rTg4510 mice showed a significant increase in LMA from 2 to 7 months of age (Fig 1D, F(4, 694) = 32.5, p < 0.0001).

### LMA in 5-month-old rTg4510 mice correlates with brain atrophy and NFT pathology

**Fig 2:**
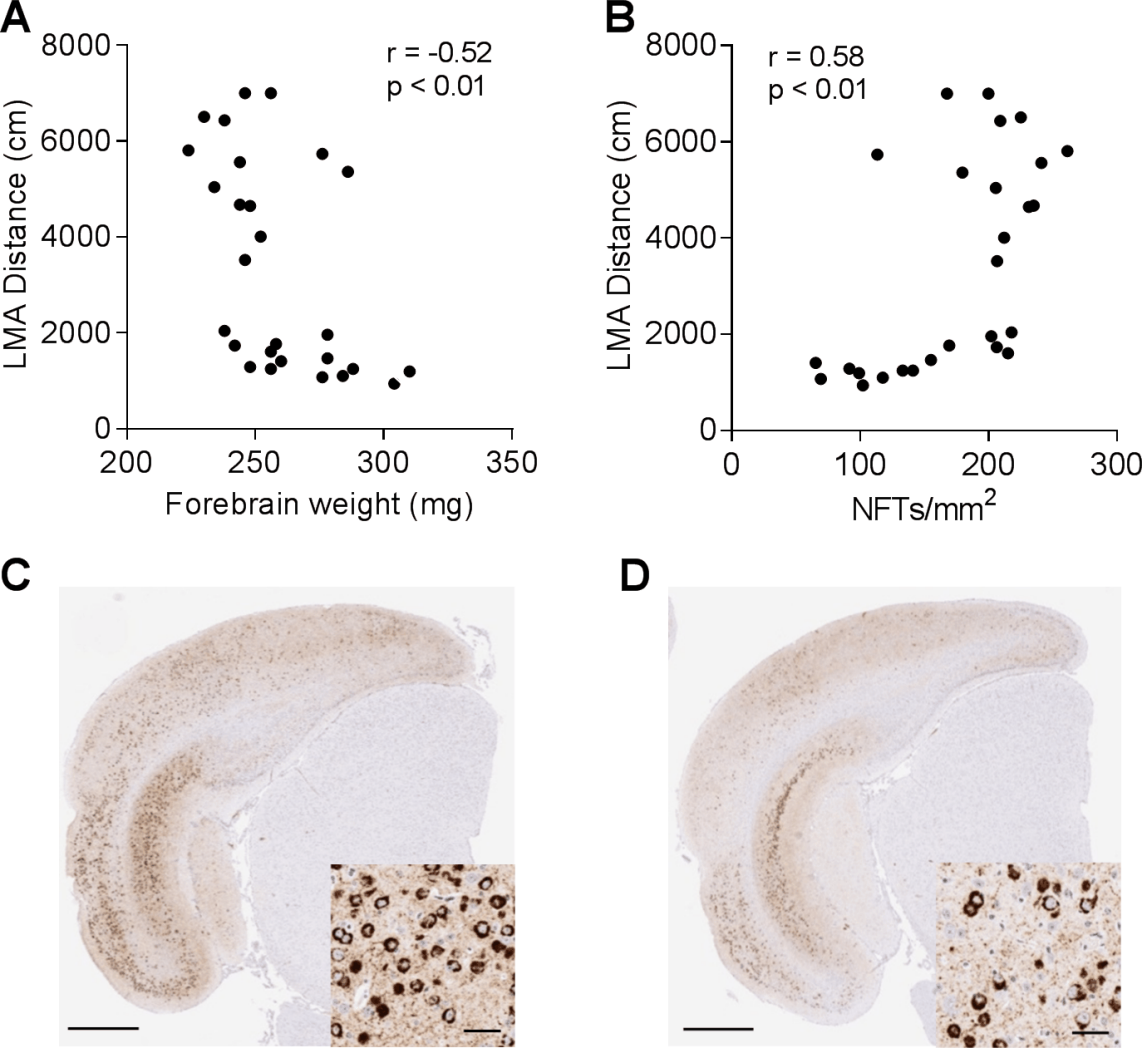
LMA in 5-month-old rTg4510 mice correlates with brain atrophy and NFT pathology. (A), Negative correlation between LMA and forebrain weight (r = -0.52, p<0.01, n = 27). (B), Positive correlation between LMA and NFT pathology in entorhinal cortex (r = 0.58, p<0.01, n = 27). (C–D), Representative images of NFT pathology in entorhinal cortex of rTg4510 mice with LMA distance > 2000 cm (C) and < 2000 cm (D). Insets, higher magnification images of NFT bearing neurons in entorhinal cortex (Scale bar, 800 μm; 20 μm in inset).

The variability of LMA between animals of the same age provided an opportunity to investigate the correlation between LMA and other pathological and biochemical endpoints. Since rTg4510 mice develop age-dependent progression of brain atrophy in forebrain structures, including hippocampus and cortex [12,13], we first examined the correlation between LMA distance and forebrain atrophy in 5-month-old rTg4510 mice. We found a significant inverse correlation between the LMA and the forebrain weight (Fig 2A, r = -0.52, p < 0.01).

To better understand the tau pathology in hyperactive rTg4510 mice, we examined the relationship between LMA and NFT counts in entorhinal cortex using nitrated tau (Tau-nY29) antibody immunostaining, which preferentially labels fibrillar tau lesions [30]. A significant correlation was found between LMA and NFT counts in entorhinal cortex (Fig 2B, r = 0.58, p < 0.01). Hyperactive rTg4510 mice with LMA distances over 2000 cm during the LMA test tended to have more tangle-bearing neurons in entorhinal cortex (Fig 2C) and hippocampus (S2 Fig) as compared to the animals with LMA distance less than 2000 cm (Fig 2D & S2 Fig).

### Correlation between LMA and brain hyperphosphorylated tau levels

To better quantitate the hyperphosphorylated tau burden in the rTg4510 model, we established an alphaLISA assay using the total tau antibody HT7 as the capture antibody and the hyperphosphorylated tau antibody PHF6 as the detection antibody.

**Fig 3:**
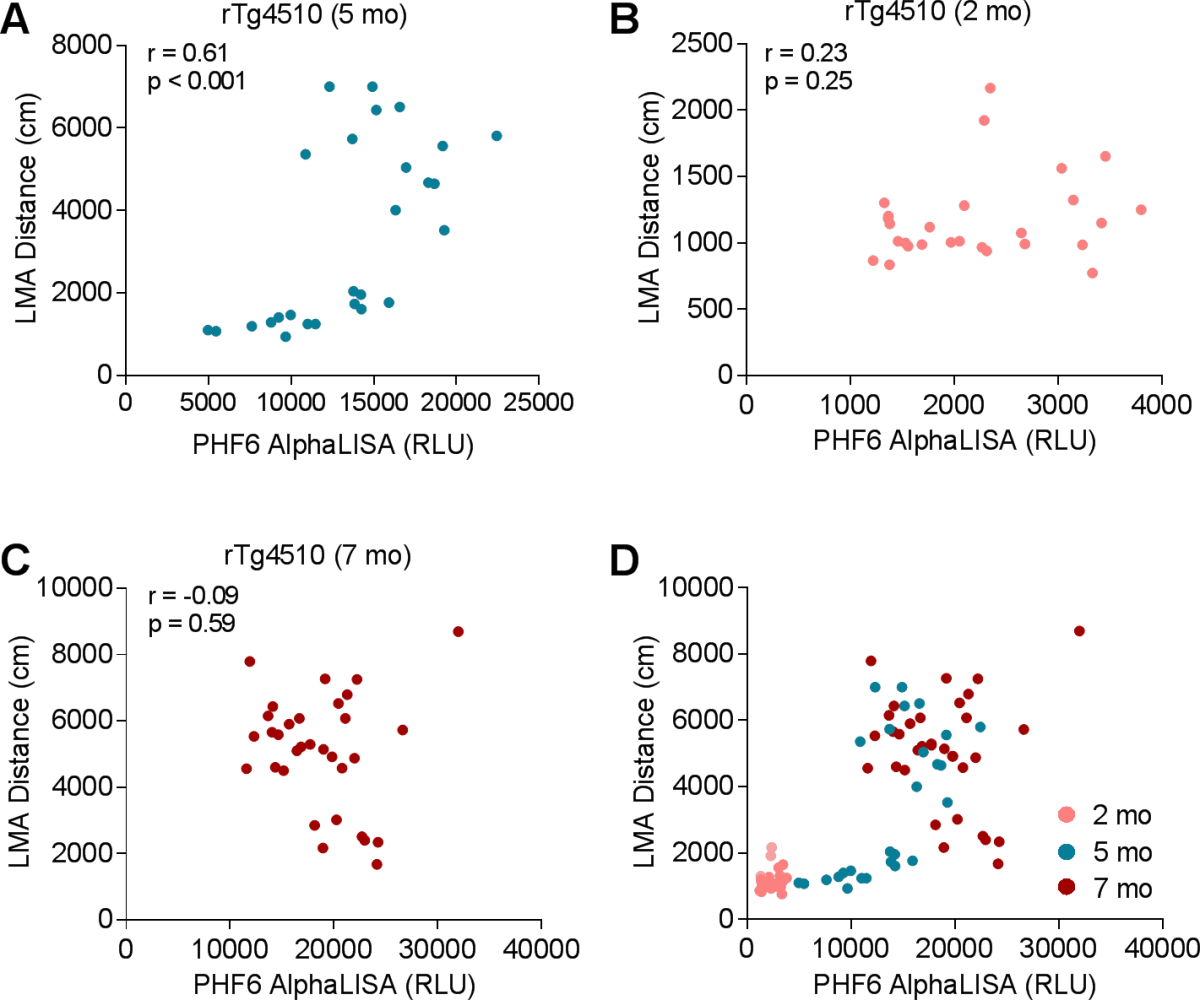
Correlation between LMA and brain hyperphosphorylated tau levels. (A) LMA positively correlated with brain hyperphosphorylated tau detected by PHF6 alphaLISA in 5-month-old rTg4510 mice (r = 0.61, p < 0.001, n = 27). (B–C) Lack of correlation between LMA and brain hyperphosphorylated tau in rTg4510 mice at 2 months of age (B, r = 0.23, p = 0.25, n = 30) and 7 months of age (C, r = -0.09, p = 0.59, n = 36). (D) Plot of LMA distance as a function of brain hyperphosphorylated tau levels at 2, 5, and 7 months of age.

The relationship between locomotor behavior and brain hyperphosphorylated tau burden was determined using correlation analysis between LMA distance and brain PHF6 alphaLISA signal at different ages. In 5-month-old rTg4510 mice, we found a significant positive correlation between LMA and PHF6 alphaLISA signal (Fig 3A, r = 0.61, p < 0.001). Interestingly, similar positive correlation was also observed in the 5-month-old mice with LMA distance less than 2000 cm (Fig 3A, r = 0.81, p < 0.001), but not in the 5-month-old mice with LMA distance over 2000 cm (Fig 3A, r = 0.4, p = 0.18). In contrast to the 5-month-old mice, 2-month-old rTg4510 mice displayed low hyperphosphorylated tau levels in the forebrain without hyperactivity, and a lack of correlation between LMA distance and PHF6 AlphaLISA signal was observed in this age group (Fig 3B, r = 0.23, p = 0.25, n = 30). At 7 months of age when both hyperactivity and hyperphosphorylated tau burden are robust, the correlation between LMA distance and PHF6 AlphaLISA signal could not be detected either (Fig 3C, r = -0.09, p = 0.59, n = 36). When the data were plotted together from 2-, 5-, and 7-month-old rTg4510 mice (Fig 3D), the combined data set illustrates 3 distinct stages of disease progression: (i) early stage as represented by 2-month-old rTg4510 mice where both brain hyperphosphorylated tau levels and LMA are low, (ii) intermediate stage as represented by a subpopulation of 5-month-old rTg4510 mice that showed significant accumulation in hyperphosphorylated tau burden without robust phenotypic change in LMA, and (iii) late stage as represented by a subpopulation of 5-month-old rTg4510 mice and most of the 7-month-old rTg4510 mice that showed robust increase in hyperactivity and lack of correlation between LMA distance and brain pathological tau burden. These findings suggest that the accumulation of pathological tau species needs to reach a threshold level to initiate the phenotypic change (locomotor hyperactivity) in the rTg4510 model.

### Doxycycline treatment prevented the progression of hyperactivity in 2-month-old rTg4510 mice, but not in 4-month-old rTg4510 mice

In order to demonstrate a causative relationship between tau and behavior, we next investigated if reducing the pathological tau levels at various stages of tau pathology could prevent or slow down the progression of hyperactivity in rTg4510 mice. Two-month-old or 4-month-old rTg4510 mice were treated with either vehicle or doxycycline diet (1 g/kg) for 4 months and monitored the progression of hyperactivity monthly. Notably, at 2 months of age, the rTg4510 mice do not have apparent pathological tau accumulation in the brain. In contrast, 4-month-old rTg4510 mice have well-established tau pathology throughout the forebrain structures [12,28].

**Fig 4:**
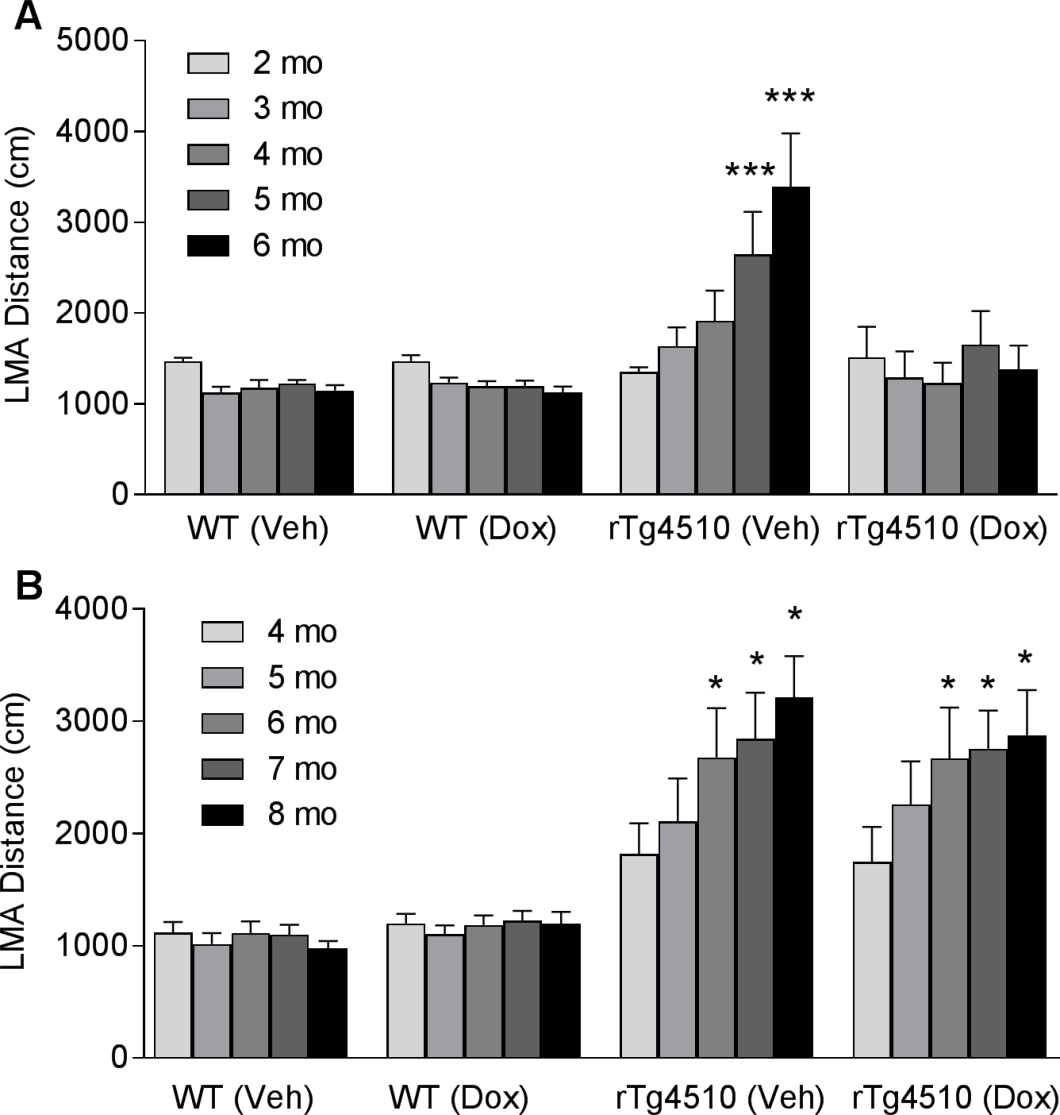
Effects of doxycycline treatment on progression of hyperactivity in rTg4510 mice are age-dependent. WT and rTg4510 mice were dosed with either normal mouse chow (Veh) or normal mouse chow containing doxycycline at 1g/kg (Dox) for 4 months starting at either 2 months of age (A) or 4 months of age (B). LMA was tested monthly after the onset of treatment to monitor the progression of hyperactivity. (n = 8 WT and 22–24 rTg4510 mice per group; ***p<0.001, Two-way ANOVA followed by Bonferroni's test compared to baseline groups).

The 2-month-old rTg4510 mice fed with vehicle diet showed a significant increase in LMA over 4 months of treatment (Fig 4A). Even at 1 month after the onset of treatment, the rTg4510 mice fed with vehicle diet already developed a trend of increase in LMA (Fig 4A). This trend became significant as treatment continued toward 5 months of age (Fig 4A, 95.7% increase, p < 0.001) and 6 months of age (Fig 4A, 150.7% increase, p < 0.001). In contrast, 2-month-old rTg4510 mice fed with doxycycline diet did not develop hyperactivity over 4 months of treatment (Fig 4A). To understand if doxycycline per se has any effects on LMA, we also included WT littermates treated with vehicle or doxycycline. We found that doxycycline treatment had no effect on LMA in WT littermates (Fig 4A).

In contrast, when treatment started at 4 months of age, doxycycline failed to prevent the progression of hyperactivity in the rTg4510 mice (Fig 4B). The rTg4510 mice fed with either vehicle or doxycycline diet showed significant increase in LMA at 6, 7, and 8 months of age (Fig 4B). Similar to the younger age group, neither doxycycline nor vehicle had any effect on LMA in 4-month-old WT mice (Fig 4B).

### Effects of doxycycline on LMA is associated with changes in brain hyperphosphorylated tau burden

**Fig 5:**
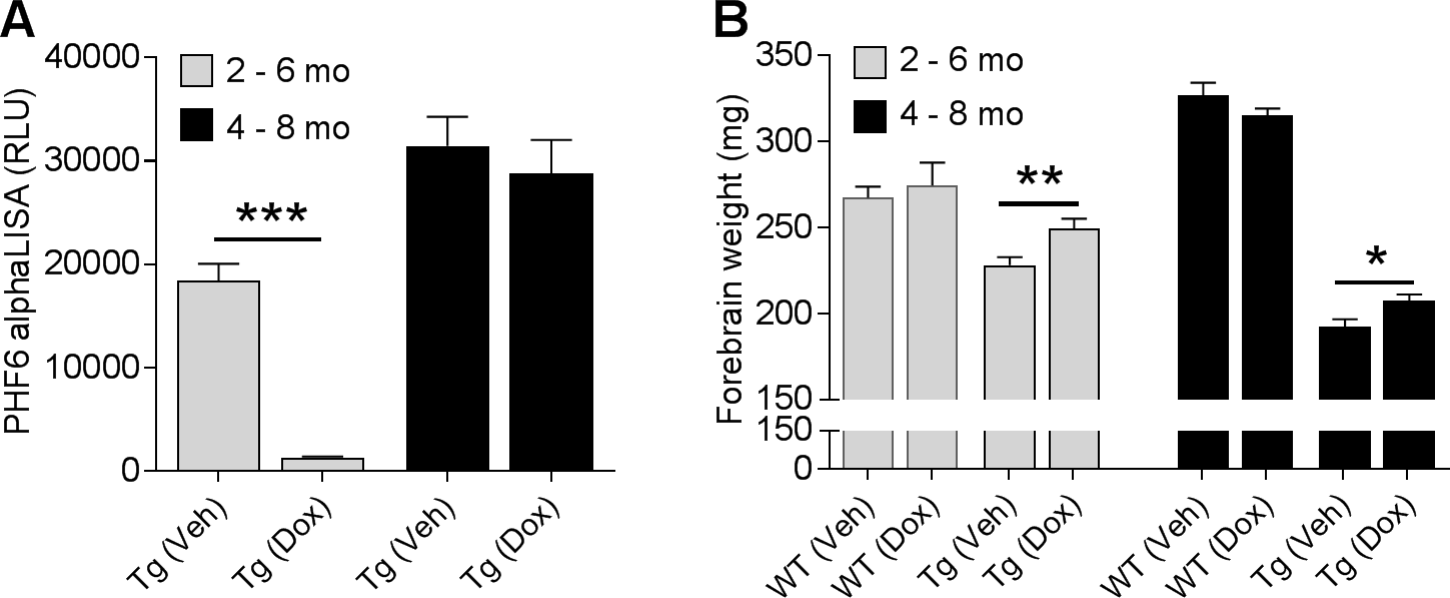
Effects of doxycycline treatment on rTg4510 disease progression. (A) Brain hyperphosphorylated tau levels were revealed by PHF6 AlphaLISA in rTg4510 mice (n = 22–24 mice/group; ***p < 0.001, t-test). (B) Effects of doxycycline treatment on forebrain weight was measured in both WT and rTg4510 mice (n = 8 WT and 22–24 rTg4510 mice per group, *p < 0.05, **p < 0.01, t-test).

The effect of reducing mutant tau expression on brain accumulation of hyperphosphorylated 64KD tau has been reported to be age-dependent in rTg4510 mice [12]. Consistent with this report, we found that doxycycline significantly reduced brain PHF6 AlphaLISA signal in the younger rTg4510 mice by 93% when treatment started at 2 months of age (Fig 5A, p < 0.001). In contrast, doxycycline had no effect on brain PHF6 signal in the older rTg4510 mice when treatment started at 4 months of age (Fig 5A, p = 0.55). The lack of effect on brain hyperphosphorylated tau in older rTg4510 mice is consistent with the finding that doxycycline failed to prevent the progression of hyperactivity when treatment started at 4 months of age (Fig 4B).

We next analyzed the forebrain atrophy upon doxycycline treatment in both rTg4510 and WT mice. Doxycycline treatment significantly reduced forebrain atrophy measured as weight in both young and old rTg4510 by 9.5% (p < 0.01) and 7.8% (p < 0.05), respectively, as compared to the rTg4510 mice treated vehicle (Fig 5B). In contrast, doxycycline treatment had no effect on forebrain weight in WT mice. These findings suggest that there is a threshold in brain degeneration which may trigger the hyperactivity in rTg4510 mice. Once the brain degeneration passes that threshold in old rTg4510 mice, slowing down the brain atrophy no longer has an effect on the hyperactivity (Fig 5B).

### Effects of OGA inhibitor Thiamet G on the progression of pathology in rTg4510 mice

**Fig 6:**
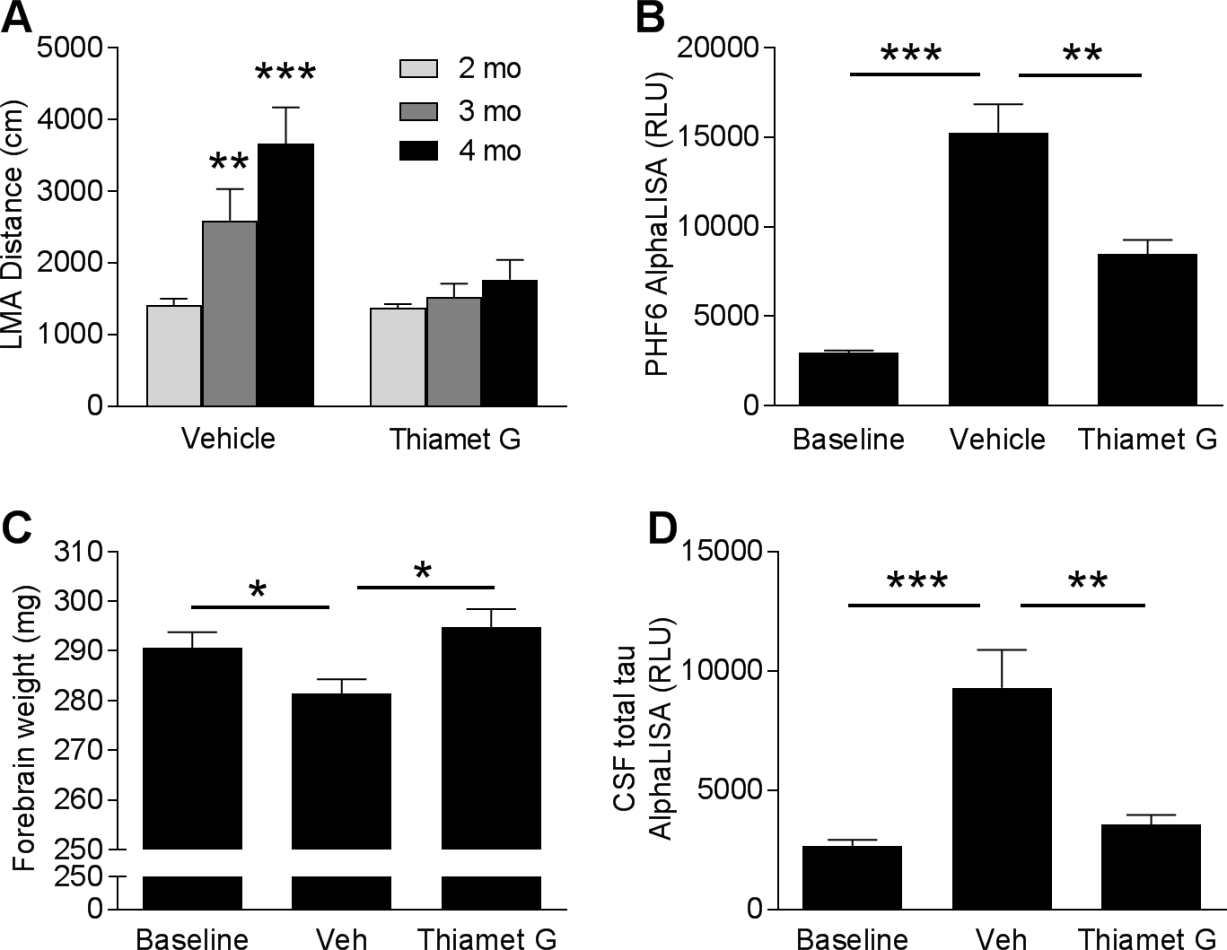
Effects of OGA inhibitor Thiamet G treatment on rTg4510 disease progression. (A) LMA was measured at the baseline (2 months of age) and monthly after the onset of treatment (n = 20–21/group; **p < 0.01, ***p < 0.001, Two-way ANOVA followed by Bonferroni's test compared to baseline groups at 2 months of age). (B–D) Thiamet G significantly reduced brain hyperphosphorylated tau levels detected by PHF6 AlphaLISA (B), prevented brain atrophy (C), and reduced CSF total tau levels (D) in rTg4510 mice (n = 21/group; *p < 0.05, **p < 0.01, ***p < 0.001, one-way ANOVA followed by Dunnett’s test compared to vehicle group).

Increases in tau O-GlcNAcylation has been shown to reduce tau aggregation and ameliorate tau pathology in animal models [24,31]. To determine whether the OGA inhibitor Thiamet G can prevent the progression of hyperactivity and tau pathology in rTg4510 mice, 2-month-old rTg4510 were treated daily with vehicle or Thiamet G at 500 mg/kg via oral gavage for 2 months. LMA was measured at 2 months of age before the dosing started and monthly thereafter to monitor the progression of hyperactivity. When given Thiamet G, the rTg4510 mice had no significant increase in LMA over the course of the study (Fig 6A). In contrast, vehicle treated animals exhibited significant age-dependent increase in LMA distance by 83.7% at 3 months of age (Fig 6A, p < 0.01) and 159.5% at 4 months of age (Fig 6A, p < 0.001). To elucidate the underlying mechanism associated with the effects of Thiamet G on hyperactivity, we analyzed the hyperphosphorylated tau levels in forebrains. Forebrain samples from a baseline group of rTg4510 mice were collected at 2 months of age before the onset of the treatment (Fig 6B). After two months, the vehicle-treated group showed a 5.2-fold increase in PHF6 AlphaLISA signal from baseline (Fig 6B, p < 0.001), whereas Thiamet G treatment significantly reduced the hyperphosphorylated tau levels by 44.4% (Fig 6B, p < 0.01). Consistent with these findings, vehicle-treated animals also exhibited a significant reduction in forebrain weight (Fig 6C, p < 0.05), whereas 2 months of treatment with Thiamet G ameliorated the brain atrophy in rTg4510 mice (Fig 6C, p < 0.05). Furthermore, CSF total tau levels were analyzed as a potential translational biomarker for clinical studies. rTg4510 mice treated with vehicle showed a significant increase in CSF total tau levels (3.5 fold from baseline; Fig 6D, p < 0.001), whereas 2 months of treatment with Thiamet G prevented the increase in CSF total tau (Fig 6D, p < 0.01). Taken together, these results demonstrate that chronic inhibition of OGA reduces the accumulation of pathological tau, slows down neuronal degeneration, and prevents the progression of hyperactivity in rTg4510 mice.

## Discussion

This study provides the first evidence that locomotor deficits in the rTg4510 model strongly correlates with the progression of brain tau pathology. Furthermore, reducing pathological tau levels with either doxycycline or Thiamet G treatment in young rTg4510 mice slows down the progression of locomotor hyperactivity and attenuates the progression of tau pathology, suggesting a further link between these two endpoints.

Locomotor activity is a fundamental component of many behavioral functions, including exploration, approach to goals, and escape from aversive situations [32]. Thus, it is critical to determine whether impairments in locomotor function in transgenic models may confound rodent cognitive tests, which rely on a mouse being able to explore objects (e.g. Novel Object Recognition), swim (e.g. Morris Water Maze), freeze (e.g. Contextual Fear Conditioning), escape (eg. Passive Avoidance) or approach a target (e.g. T-maze). Locomotor deficits were initially observed in tau transgenic models in which PrP or Thy1 promotor drives mutant tau expression [6,11]. In those animal models, pathological tau is widely observed in motor neurons within brainstem and spinal cord regions. In contrast, mutant tau expression in rTg4510 mice is driven by the forebrain specific CamKIIα promotor to avoid the pathology in brainstem and spinal cord regions and associated locomotor deficits. However, forebrain circuits also play a role in modulating locomotor function [32,33]. For example, hippocampal lesions render rats hyperactive in familiar and non-familiar environments [34–36], which might reflect loss of inhibitory control over accumbal dopamine transmission [37]. Moreover, increased locomotor activity has also been observed after entorhinal cortex lesion [34], which may reflect dysfunction in spatial information processing. Consistent with these findings, the current study revealed severe brain atrophy associated with tau pathology in both hippocampus and entorhinal cortex in rTg4510 mice. Therefore, the neuronal degeneration in these brain regions may underlie the hyperactivity phenotype.

Hyperactivity in rTg4510 mice could be associated with anxiety-like behavior as impaired anxiety has been observed in several lines of tau and APP transgenic mice [38–41]. In the current study, although 5-month-old rTg4510 mice exhibited a robust increase in LMA, the percentage of center region activity of the transgenic mice is comparable to the WT littermates. As reduced center region activity is generally associated with an anxiety-like phenotype [29], these results suggest a lack of anxiety-like behavior in rTg4510 mice at this age. Of course, it is possible that older rTg4510 mice may develop anxiety-like behaviors as tau pathology and neurodegeneration further spreads, for example to the amygdala. Future studies will need to address this possibility.

It is reasonable to speculate that the hyperactivity observed in rTg4510 mice might represent a model for the wandering behavior observed in dementia patients. Wandering behavior is one of the most frequently encountered dementia-related behavioral disturbances and has been associated with the higher morbidity and mortality [18,42]. Although the etiology of wandering is not clear, there is evidence showing that AD patients that wander have reduced frontotemporal glucose utilization, suggesting that neuronal degeneration in frontotemporal regions may contribute to this type of behavioral disturbance [43]. In addition, behavioral disturbances in FTDP-17 patients are often characterized by disinhibition associated with inappropriate behavior and poor impulse control [44]. These symptoms might also be relevant to the hyperactivity observed in rTg4510 mice.

Although the number of NFTs has been found to correlate closely to the severity of cognitive deficits in AD, the exact pathological tau species that contributes to the behavioral impairments in tauopathy patients is not clear [12]. Here we show that suppression of mutant tau from 2 to 6 months not only prevented the progression of hyperactivity in rTg4510 mice but also halts the accumulation of hyperphosphorylated 64 KD tau detected by PHF6 alphaLISA. In contrast, when doxycycline was given to 4-month-old rTg4510 mice after tau pathology was well established, neither hyperphosphorylated tau nor progression of hyperactivity was affected by the treatment. Thus, progression of hyperactivity correlates with hyperphosphorylated 64 KD tau levels. These findings are consistent with a previous report showing that the effects of mutant tau suppression on the progression of brain tau pathology are age-dependent in the rTg4510 model [12]. Interestingly, that same report showed that suppression of mutant tau at 5.5 months of age did ameliorates certain cognitive deficits [12], further suggesting the importance of studying multiple endpoints and each endpoint’s response as a function of treatment initiation.

Chronic treatment with the OGA inhibitor Thiamet G has been shown to ameliorate tau pathology and prevent neurodegeneration in several tau transgenic models [24,31]. Here we found that Thiamet G treatment starting at 2 months of age prevented the progression of hyperactivity in rTg4510 mice. Consistent with previous reports, Thiamet G also reduced hyperphosphorylated tau and prevented brain atrophy in rTg4510 mice. The current study only explored the efficacy of OGA inhibitor Thiamet G in young rTg4510 mice given that mutant tau suppression with doxycycline failed to halt the pathological tau accumulation and the progression of hyperactivity when treatment started at 4 months of age. However, the mechanism of action for OGA inhibitor is likely due to the inhibition of tau aggregation [24], which is distinct from that of doxycycline treatment. Therefore, future studies should also investigate whether OGA inhibitor treatment can ameliorate disease progression in aged rTg4510 mice. Overall, these results suggest that OGA is a potential target for ameliorating neurodegeneration and the associated behavioral impairments in patients with tauopathies.

In conclusion, our results provide important insight into the relationship between hyperactivity and brain tau pathology in rTg4510 model. These findings may help to better understand the mechanism underling the behavioral disturbances in tauopathies.

## Supporting information

S1 Fig PHF6 AlphaLISA signal correlates with hyperphosphorylated 64KD tau in rTg4510 mouse brain. (A) Western blot analysis of total tau and hyperphosphorylated 64KD tau in brain homogenates from 5-month-old rTg4510 mice. Total tau and hyperphosphorylated 64KD tau were detected by HT7 and PHF6 antibodies, respectively. The position of the 55 KD and 64 KD tau species are indicated. β-actin was used as a loading control. (B) 64KD tau in PHF6 Weston Blot correlates with 64KD tau in HT7 Weston Blot. (C–E) PHF6 AlphaLISA signal shows a strong correlation with 64KD tau in PHF6 Western Blot (C), 64 KD tau in HT7 Western Blot (D), and NFT levels in entorhinal cortex (E), (n = 27 mice).
(TIF)

S2 Fig Representative images of NFT pathology in hippocampus of rTg4510 mice with average LMA distance >2000 cm (A-C) and < 2000 cm (D-F). Insets, higher magnification images of NFT bearing neurons in CA1 and CA3 regions of hyperactive rTg4510 mice (B-C) and the mice with normal LMA (E-F) (Scale bar, 500 μm; 20 μm in inset).
(TIF)
