# Supplementary figures and images for: Early intervention of tau pathology prevents behavioral changes in the rTg4510 mouse model of tauopathy
Source: PLoS One. 2018 Apr 6;13(4):e0195486. doi: 10.1371/journal.pone.0195486 (PMC5889169; doi:10.1371/journal.pone.0195486)

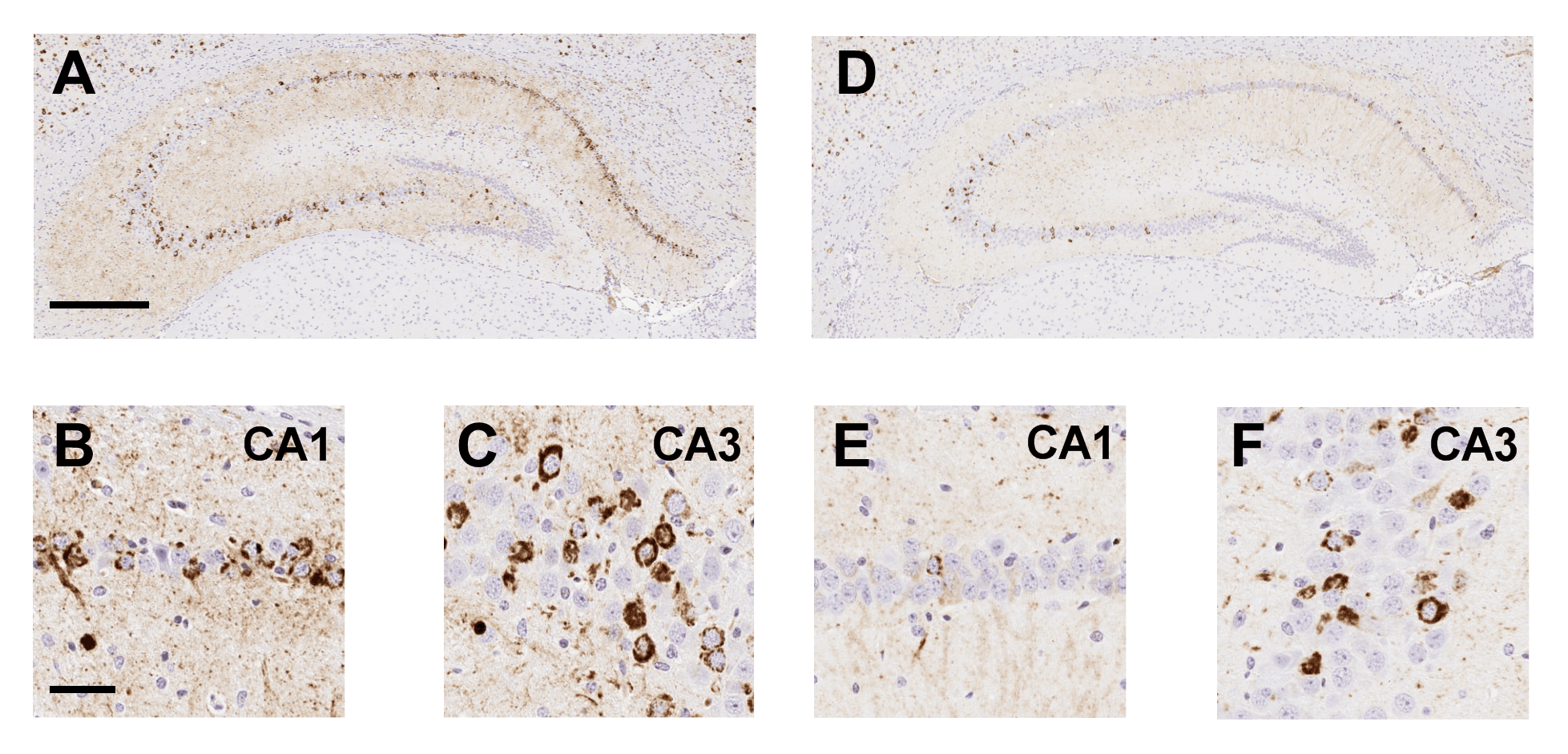

Supplement: S2 Fig — Representative images of NFT pathology in hippocampus of rTg4510 mice with average LMA distance >2000 cm (A-C) and < 2000 cm (D-F). Insets, higher magnification images of NFT bearing neurons in CA1 and CA3 regions of hyperactive rTg4510 mice (B-C) and the mice with normal LMA (E-F) (Scale bar, 500 μm; 20 μm in inset). (TIF) [file pone.0195486.s002.tif]
